# Postoperative Atrial Fibrillation Occurring After Coronary Bypass Graft Surgery

**DOI:** 10.21470/1678-9741-2022-0223

**Published:** 2023

**Authors:** Ufuk Aydın, Mesut Engin, Gamze Fidan Cebeci, Yusuf Ata

**Affiliations:** 1 Department of Cardiovascular Surgery, University of Health Sciences, Bursa Yuksek Ihtisas Training and Research Hospital, Yildirim, Bursa, Turkey. E-mail: mesut_kvc_cor@hotmail.com; 2 Department of Nursing, University of Health Sciences, Bursa Yuksek Ihtisas Training and Research Hospital, Yildirim, Bursa, Turkey

Dear Editor,

We have read the article by Cui et al.^[1]^ entitled “New-Onset Post-Operative Atrial Fibrillation in Patients Undergoing Coronary Artery Bypass Grafting Surgery - A Retrospective Case-Control Study” with great interest. First of all, we congratulate the authors for their valuable contribution to the literature. However, we would like to discuss some points about postoperative atrial fibrillation (PoAF).

PoAF occurring after coronary artery bypass grafting (CABG) is detected at rates of up to 50%. PoAF is a potentially lethal and morbid complication that prolongs hospitalizations and increases hospital costs^[2,3]^. In this current study, 233 patients who underwent off-pump CABG were included. Also, exclusion criteria were determined as patients with a known history of atrial fibrillation (AF), supraventricular arrhythmias, no coronary angiography result, and use of anti-arrhythmic drugs other than beta-blockers, digoxin, and calcium-channel blockers. We could not find any information about chronic obstructive pulmonary disease and thyroid hormone data in the study. Were patients with these problems included in the study? It is known that these problems are related to the development of AF^[4,5]^. In addition, in the study, how the echocardiographic evaluation was performed was given in detail. Regarding this situation, the definitions of “Postoperative right and left atrial enlargement” were used as data in Table 2. How many millimeters above is considered an enlargement?

In our clinical practice, PoAF is frequently seen on postoperative days 2-4. In the Cui et al.^[1]^ study, PoAF was frequently seen on day 0. Our clinic is also a high-volume clinic, and we see very little PoAF on day 0. What could be the reason for the most common day 0 for PoAF in this study? Is the situation similar in other open heart operations in that clinic? Our thoughts seem to be in line with the literature as well^[6,7]^.

One of the important issues in PoAF studies is how to follow the rhythm in the postoperative period. In Cui et al.^[1]^ study, continuous electrocardiography follow-up was performed after the operation in the intensive care unit. Also, standard 12-lead electrocardiography was recorded in the ward until hospital discharge. However, patients were not monitored continuously in the ward. Could PoAF have been missed in some patients who did not describe complaints?

Also, in this study, the authors stated that after univariate associations analysis by logistic regression modeling, all significant variables at a nominal two-tailed *P*≤0.20 were then entered into multivariable logistic models using a combination stepwise selection method. Here, the patient groups were determined as 75 patients who developed PoAF and 158 patients who did not. Seventeen variables were included in the multivariable analysis. Could this be the cause of overfitting?^[8]^

Finally, we would like to express an opinion on the variables of hospital and intensive care length of stay included in the multivariate analysis. Do Cui et al.^[1]^ think PoAF is seen more frequently in patients with longer hospital and intensive care unit stays? Or are patients with PoAF more hospitalized?
